# Exploring the Question: “Does Empathy Work in the Same Way in Online and In-Person Therapeutic Settings?”

**DOI:** 10.3389/fpsyg.2021.671790

**Published:** 2021-09-21

**Authors:** Raffaele Sperandeo, Valeria Cioffi, Lucia Luciana Mosca, Teresa Longobardi, Enrico Moretto, Yari Mirko Alfano, Cristiano Scandurra, Benedetta Muzii, Daniela Cantone, Carmela Guerriera, Marco Architravo, Nelson Mauro Maldonato

**Affiliations:** ^1^Postgraduate School of Integrated Gestalt Psychotherapy, Torre Annunziata, Italy; ^2^Department of Neurosciences and Reproductive and Odontostomatological Sciences, University of Naples Federico II, Naples, Italy; ^3^Department of Humanistic Studies, University of Naples Federico II, Naples, Italy; ^4^Department of Psychology, University of Campania Luigi Vanvitelli, Caserta, Italy

**Keywords:** video conferencing psychotherapy, digital empathy, electronic-based therapy, telepsychology, remote clinical psychology, online therapeutic settings, in-person therapeutic settings

## Abstract

Providing remote psychotherapy using technology is a growing practice, especially since the outbreak of the COVID-19 pandemic. Even if in numerous studies video conferencing psychotherapy (VCP) was found to be clinically effective, some doubts continue to exist about how the psychotherapeutic alliance works in the online setting, and the characteristics of the empathic process are still poorly understood. This is an exploratory study aimed at analyzing the degree of empathy between the psychotherapist and client pair, and the degree of support perceived by the client who shall be referred to as the patient interchangeably in this study, comparing the sessions in person with those online, during the current pandemic, in order to discriminate the impact of empathy in the digital setting. The sample analyzed was composed of 23 patients with different severity of pathology engaged in online and in-person therapeutic sessions with five psychotherapists of different theoretical leanings. The scores of the support and empathy scale, obtained by both members of the psychotherapeutic couple in the two settings, were analyzed and compared. The test used belongs to an Italian adaptation of the Empathic Understanding (EU) of the Relationship Inventory. What emerged from comparing the scores was interesting: Unlike the psychotherapists, the patients perceived their therapists as significantly more empathic and supportive in the remote setting. These are rather important data, because the literature documents that client empathic perception measures represent a more accurate measure of the empathic relationship and, in general, can predict a good treatment outcome. Although these results need further investigation, they represent an important contribution in filling the scientific gap in the understanding of digital empathy. Also, this study provides new insights for future research on the characteristics and impact empathy has on the practice of remote psychotherapy.

## Introduction

Since the day the World Health Organization declared the new SARS-CoV-2 coronavirus as a pandemic because of its global outbreak, unprecedented changes have happened in the personal and professional activities of the whole Italian population (Di Corrado et al., 2020). In this challenging period, the coronavirus has not been the only health risk, since everyone has to continue to manage stress (Maldonato et al., 2020) and take care of their personal, physical, and psychological wellbeing. For this reason, a lot of health specialists have been able to continue working online, assisting their patients from home too (Reilly et al., 2020). Among them are a lot of psychotherapists who have been able to carry on with their psychotherapy sessions remotely, through video conferencing psychotherapy (VCP), thus ensuring health benefits (Cioffi et al., 2020).

Since the beginning of this century, international studies have analyzed the benefits, possibilities, limits, and faults of various online psychological interventions (Cipolletta et al., 2018); they highlighted that VCP can be practicable, clinically effective, and suitable to patients. VPC has been used in a multiplicity of therapeutic plans and with different kinds of patients, it is generally associated with good user satisfaction, and it is found to have clinical outcomes comparable to traditional frontal psychotherapy (Backhaus et al., 2012; Berryhill et al., 2019a,b; Dolce et al., 2020).

Video conferencing psychotherapy has a lot of advantages, first of all, it can reduce and almost eliminate the distance between one and another, which is an important factor for those who live in under-served regions; moreover, it makes it possible to overcome many challenges, for example, time restraints, scheduling troubles, and other customer inconveniences regarding the concern of social stigma in seeking care, enabling the latter to overcome these difficulties by engaging with professional services in the privacy of their home (Sperandeo et al., 2020). VCP is an opportunity for those organizations that serve geographically disperse or isolated populations for different reasons. It is also useful for people with special needs, with mobility problems for different reasons, with specific psychic disorders limiting travel, with socialization problems, or with serious pathologies (Cioffi et al., 2020). However, some doubts continue to exist about VCP use and usefulness.

One of these has to do with some debates on the possibility to form a satisfactory working alliance within the psychotherapist-client couple when psychotherapy is provided through such a medium.

Evidence coming from a systematic literature review demonstrated both an adequate working alliance and suitable outcome for VCP; while two recent meta-analyses found that the working alliance in VCP seemed to not be as good as that which is obtained in face-to-face sessions, while that difference had nothing to do with the distinctive pathologies of patients (Norwood et al., 2018).

The hypothesis that therapist empathy is a key element in the process of change in psychotherapy has ancient roots. The results of a meta-analysis on the relationship between therapist empathy and client outcome showed that empathy is a reasonably strong predictor of therapy outcome (Elliott et al., 2018). Consequently, empathy is certainly one of the fundamental factors capable of determining an adequate working alliance between psychotherapist and client within a session, regardless of the psychotherapeutic approach (Elliott et al., 2011). The fundamental role of empathy in patient care and the patient-psychotherapist relationship is well recognized in literature (Feller and Cottone, 2003; Nascivera et al., 2018).

Empathy is a complex construct and there are lots of definitions of it according to the various disciplines or the author’s backgrounds. A definition that takes into account the different definitions comes from Batson (2009), who described empathy as a psychological state, that is at the same time a skill and a process, of which there are eight phenomena parts (see Supplementary Appendix Table 1).

Starting from this complex vision of empathy and how it is able to influence the therapeutic process in the face-to-face sessions, we asked ourselves if the latter had the same characteristics in online sessions.

The advent of digital information and communication technology has converted human interactions into digital conversations in which people can instantly share thoughts, feelings, and behaviors through digital channels. The concept of digital empathy has its roots in these changes in the way human beings interact during the digital age. In particular, Terry and Cain (2016) gave the following definition of the concept: “*traditional empathic characteristics such as concern and caring for others expressed through computer-mediated communications*.” Then Friesem (2016b) underlined how digital empathy pushed us to a broader understanding of traditional empathy, in order to be able to understand its expression in the digital universe. This latter author, taking up the model of Batson’s “*eight empathy phenomena*,” deepens and further describes the characteristics of digital empathy: “*digital empathy explores the ability to analyze and evaluate another’s internal state (empathy accuracy), have a sense of identity and agency (self-empathy), recognize, understand and predict other’s thoughts and emotions (cognitive empathy), feel what others feel (affective empathy), role play (imaginative empathy), and be compassionate to others (empathic concern) via digital media*” (Friesem, 2016a).

There are three main categories to measure empathy in psychotherapy settings: (a) self-reports filled out by the patients, the psychotherapist, or outside observer; (b) outside observer’s assessments through specific assessment grids for evaluating recorded psychotherapy sessions; and (c) measurements of psychophysiological response variations (skin conductance, oxygen saturation, blood pressure, and heart rate) (Messina et al., 2013). Of these kinds of assessments, the empathy perceived by the patients was considered the best predictor of psychotherapy outcome (Grummon, 1972).

Among the most common instruments utilized to assess perceived empathy in psychotherapy, there is the Empathic Understanding (EU) of the Relationship Inventory (Barrett-Lennard, 1986), of which the Italian version is the Scale dell’Empatia Percepita (SEP; Messina et al., 2013). This Inventory gives an evaluation of empathy based on Carl Rogers’ theories on therapeutic helping and person-centered therapy (Meador and Rogers, 1984). The Italian version (SEP) contains the form for the client (SEP-A), to measure the empathy perceived by the client during the session; and the form for the psychotherapist (SEP-M), to evaluate the empathy that the psychotherapist thinks they have communicated to his/her client during the session. In research for validating the Italian version, it has been shown that SEP-A reflects sensory empathy while SEP-M reflects more complex affective empathy relating to emotion sharing and interpersonal relationships (Messina et al., 2013).

Beginning from these reflections about empathy and digital empathy, we wondered if empathy works in the same way in online and in-person therapeutic settings, and what the differences between the treatment and the outcomes are.

## Materials and Methods

This is an exploratory study aimed at analyzing the level of affective attunement and more precisely the degree of empathy among the members of the psychotherapist-client dyad, as well as the degree of support perceived by the patient, comparing the sessions in-person with those online, during the period of lockdown necessitated by the COVID-19 pandemic, in order to discriminate the specific characteristics of digital empathy.

### Participants

The sample analyzed is composed of five psychotherapists (2 men and 3 women) of different theoretical orientations (psychoanalysts, transactional analysts, and Gestaltists) and 23 patients (4 men and 19 women) with different severity of the pathology (11 without current psychic disorders, 7 with mild psychic disorders, and 5 with moderate psychic disorders) engaged in weekly or fortnightly psychotherapeutic treatments.

### Data Collection and Procedures

This is an open study due to the small number of subjects included. While the topic of the psychotherapeutic alliance in online settings is well studied, so there is a good amount of research on it, that of empathy (which is one of the components of the therapeutic alliance) is still a little-explored topic. We aimed to explore how empathy works in online settings, for this reason, we did not select the sample based on specific parameters to prevent our unconfirmed hypotheses from influencing the results. We kept the open observation typical of the exploratory survey without selecting specific inclusion parameters to ensure sample variability.

We opted to include in the study psychotherapists with various leanings who, in this period of the pandemic, were carrying out both face-to-face and remote treatments in their offices. The study is still open and the increase in therapists included in the sample will allow us, as soon as an adequate number of subjects is reached, to highlight any characteristics of the therapists related to the empathy experience.

Five psychotherapists from three different approaches voluntarily joined the study, they identified among their patients those who had voluntarily agreed to participate in the study (for a total of 23 subjects), informing them on the modalities of the study and asking them to sign the informed consent.

In particular, the psychotherapists identified among their patients those with whom they had a good alliance and were in an advanced stage of therapy (at least more than 3 months), this was to avoid that being included in the study could lead patients to drop out.

In this way, we had 23 dyads, all engaged in a healing relationship with the typical characteristics of the different psychotherapeutic models.

All online sessions took place via Skype or WhatsApp video call. Overall, 50% of patients used their PC, 15% used tablets, and 35% used a smartphone. While 88.2% of psychotherapists used their PC, 23.5% used tablets, and only one of them used a smartphone. A total of 70% of patients affirmed they had the online sessions alone from home or an office, while the remainder had the sessions in the presence of other people from home or at an office. Though 40% of psychotherapists affirmed they had the online sessions alone from home or an office, therefore most of them were at their home or office with other people in other rooms (see Supplementary Appendix Figure 1).

At the end of each psychotherapy session and for a consecutive number of four sessions, each couple (psychotherapist-patient) completed online an Italian adaptation of the Barrett-Lennard Relationship Inventory (version 3—developed by Godfrey T. Barrett-Lennard) and the Empathy and Support Scale (ESS). We have not selected specific psychotherapeutic interventions precisely to allow the breadth of perspectives of an open study. Since the experience of empathy perceived by the therapist and the patient is documented to be a phenomenon closely related to each separate session (Elliot et al., 2002), we randomly administered the test to the patient/psychotherapist dyad depending on the phase of the treatment. This allowed us to compare empathic perception in individual sessions (even if these data are not yet sufficiently confirmed from a numerical point of view, and for this reason they have not been presented) highlighting that even the same patient/therapist dyads present differences in perception of empathy in remote sessions compared to those in person.

The same patients had both online and face-to-face sessions, randomly, in accordance with their possibilities and needs. We did not give any indications regarding the alternation of sessions (in person or remote), but we simply observed the natural alternation that occurred between the dyads, in order to respect the naturalness of the therapeutic process, which is already tried by the difficulties of direct contact caused by the pandemic. Probably, the variable “personal predisposition” to the use of technological devices influenced the choice of the online setting. Moreover, for some people, the anguish of contracting the virus was a reason for preferring the online setting. Additionally, it must be said that this pandemic has also represented a sort of opportunity for some people who tended to be inconstant in their psychotherapeutic paths because they were very busy. What we mean is that for many patients the online setting has represented an opportunity to reconcile the various commitments that were often an impediment to go to the psychotherapy site.

A total of 72 sessions (33 in person and 39 online) were collected from November 2020 to January 2021. The averages scores obtained at the 72 sessions were compared, dividing and matching the sessions into two groups (one group of face-to-face sessions and the second group of online sessions).

### Measures

For the assessment of psychopathology of the patients, at the first session, the psychotherapist filled out the Comprehensive Psychopathological Rating Scale (CPRS; Åsberg et al., 1978). The CPRS consists of 40 items that explore the psychopathology reported by the patient and 25 that refer to the psychopathology observable during the interview. At the end, the evaluator must express a judgment on the overall seriousness of the clinical condition and on the degree of reliability of the information collected. The items are rated on a 4-point Likert scale, from 0 to 3. For each item, the severity levels are carefully defined; three dimensions contribute to their definition: severity, frequency, and duration.

At the end of every session, for measuring the degree of support and empathy perceived by both the client and the therapist, as well as their concordance, the Italian version of the Barrett-Lennard Relationship Inventory (version 3—developed by Godfrey T. Barrett-Lennard) was used (Barrett-Lennard, 2015). This Inventory has two forms: Other Toward Self form (40 items) aiming to evaluate the empathy perceived by the client during the session; and the Myself to Others form (40 items) to evaluate the empathy that the psychotherapist thinks they have communicated to his/her client. The items are rated on a 3-point Likert scale. This inventory explores the degree of empathy and support through two subscales, with one having items formulated positively and the other one having items formulated negatively. This inventory was created to be adapted to specific contexts of use, for this reason, we have developed an Italian adaptation, the ESS, organized over 28 items (14 positively formulated, which form the Empathy and Support Positive Subscale-ESPS, and 14 negatively formulated, which form the Empathy and Support Positive Subscale-ESNS) for the client version (ESS-C) and 28 items (14 positively formulated, which form the Empathy and Support Positive Subscale-ESPS, and 14 negatively formulated, which form the Empathy and Support Positive subscale-ESNS) for that of the psychotherapist (ESS-P) (as shown in the Supplementary Appendix).

### Analysis

The collected data were analyzed through the Statistical Package for the Social Sciences (SPSS) by performing descriptive statistics to show the qualitative and quantitative composition of the examined sample. Comparisons between the averages of the scores obtained from the two groups of subjects (online treated and in-person treated) to the empathy and support subscales were performed with Student’s *t*-test. Comparisons between the concordance between patients and therapists were performed with the χ^2^ test applied to the observations made in-person and online.

## Results

The perception of empathy and support was evaluated in parallel in the two members (patient and therapist) of the 24 therapeutic couples after four consecutive sessions. Overall, empathy and support perceived in parallel by patient and therapist were assessed after 72 therapy sessions, 39 of which were carried out remotely and 33 in person. Most of the therapeutic dyads that conducted three consecutive therapy sessions used only one type of setting (remote or in-person), three couples alternated between the setting in person and the remote one. The severity of current psychopathology in the patients was assessed by the therapist after the first of the sessions analyzed by applying the CPRS (see Supplementary Appendix Table 2).

The scores obtained by therapists and patients in the subscales test evaluating the perception of empathy and support after the face-to-face sessions were compared with those obtained after the remote sessions by taking the Student’s *t*-test. Therapists do not show significant differences in perceiving themselves capable of offering empathy and support in the two types of settings evaluated. Patients, on the other hand, perceive therapists to be significantly more empathic and supportive in the remote setting (see Supplementary Appendix Table 3).

The two subscales allow an assessment of the agreement between patient and therapist. Overall, 980 observations were made for both subscales. With regard to the ESPS, the percentage of concordance of the responses between patient and therapist in remote sessions is 70.7%, significantly higher than the percentage of agreement (62.9%) found in face-to-face sessions. Similarly, for the ESNS, the percentage of agreement in remote sessions (82.9%) is significantly higher than that detected in-person (71.7%) (see Supplementary Appendix Table 4).

These data have no significant correlation with the psychopathological aspects of the patients.

## Discussion

This study was the second phase of a larger research in which the first step was to evaluate the degree of satisfaction of an Italian sample of psychotherapists in the use of VCP during the COVID-19 emergency (Cioffi et al., 2020). In that previous phase, the attention to the relational aspects, according to the theoretical and methodological background of the psychotherapist, was found to be an element capable of fostering the therapist’s perceived satisfaction using VCP. For this reason, in this second phase, we hypothesized that the level of affective attunement and more widely the degree of empathy between the members of the psychotherapist-client dyad has specific characteristics and represents an efficacy factor for the success of the treatment. However, due to the small number of participants, we are still in an exploratory phase of the results.

In particular, during the current pandemic, the degree of empathy among the members of the psychotherapist-client dyad, as well as the degree of support perceived by the patient during the session, were analyzed. Successively the sessions in-person were compared with those online, in order to discriminate the specific characteristics of digital empathy. What emerged from the Student’s *t*-test, comparing the scores obtained by therapists and patients to the two ESS subscales after both the face-to-face sessions and the remote sessions, was really amazing. The therapists did not show significant differences in perceiving themselves as capable of offering empathy and support in the two types of settings evaluated. Patients, on the other hand, perceived therapists to be significantly more empathic and supportive in the remote setting.

This surprising finding is consistent with the results of another online group psychotherapy study (Weinberg, 2021). The authors pointed out that some group members may benefit from online groups more than in person, although they affirmed the online format is not for everyone. These pieces of evidence reinforce what has already been demonstrated about the effectiveness of this psychotherapeutic format and how the therapeutic alliance seems to be achievable also online.

The “personal predisposition” variable is certainly important and yet we believe that this was a self-selection feature of the field because many colleagues with a little predisposition to the use of telematics tools have not initiated treatments in a remote setting. Furthermore, in our previous study (Cioffi et al., 2020), we found that the therapists who liked and felt the effectiveness of the intervention at a distance were mainly those who had previously used this technique. Our previous findings are confirmed by other pieces of evidence that suggest psychotherapists’ attitudes toward online psychotherapy are influenced by their past experiences (such as clinical experience and previous online psychotherapy experience) as well as their transition experience during the pandemic and their geographic location (Békés and Aafjes-van Doorn, 2020).

In another study that evaluated the effects of the telepsychological format on empathic accuracy and therapeutic alliance, there were no statistically significant differences between the conditions on the therapist’s empathic accuracy or the therapeutic alliance. Attitudes toward telepsychology and empathic accuracy were both significant predictors of alliance in telepsychology delivery formats. The authors also argued that empathic accuracy may be a more important process for clients receiving services in the telepsychological format, so further investigation is needed (Reese et al., 2016).

We, on the other hand, focused on the subjective experience of the patient, as it is documented in the literature that the client’s empathic perception measures represented a more accurate measure of the empathic relationship and, in general, they were able to predict a good outcome client (Elliott et al., 2018). Already several studies had overwhelmingly supported the idea that the therapeutic alliance could be developed during VCP, highlighting how clients, with different diagnoses, valued bonding and presence at least as strongly as face-to-face (Simpson and Reid, 2014). In another study coming from telemedicine, no differences were found between telemedicine and in-person visits in the patient’s perception of the physician’s empathy in acute stroke care. Therefore, the authors concluded that, in a telemedicine meeting, in the context of acute stroke care, empathy does not require physical touch or physical proximity to be transmitted, but can also be transmitted only through facial expression, vocal intonation, and attentive participation (Cheshire et al., 2020).

During the current pandemic, recent studies advise that VCP can lead to a renewal of the concept of the therapeutic relationship, i.e., it offers a powerful pathway for clients to experience improved chances for self-expression, connecting, and closeness. In particular, this presupposes that, during the VCP, people would find the chance to have a more neutral psychotherapeutic “place,” where they could have more occasions for self-awareness, creative experience, and collaboration and at the same time feel they were more capable of acting on their own experience (Simpson et al., 2020).

In our study, the fact that patients feel psychotherapists are more empathetic and more capable of providing support in the online sessions cannot fail to take into account the particular moment due to the pandemic. In fact, due to the pandemic, face-to-face sessions do not enjoy the same comfort as online sessions and many patients say that. Currently, the in-person sessions are carried out with masks, plexiglass dividers, and the safety distances are strongly maintained. This is not the usual psychotherapy setting. Especially the patients who were already in treatment know the difference, they know that due to the pandemic, the psychotherapy setting has had to undergo changes to the detriment of comfort. Therefore, in agreement with what was found by Cheshire et al. (2020), we can affirm that facial expression, vocal intonation, and attentive participation are very important variables able to condition and influence the empathy perceived by the patients. In this sense, we could say that the patients in the study feel much more understood and supported by their psychotherapists during the online sessions because they can perceive facial expressions, intonations of voice, and compassionate attention of their psychotherapists, i.e., even though they speak through the PC screen, they do it without any security filters.

Moreover, to explain this result we can tap into the differences between face-to-face empathy and empathy mediated by a digital device. Authors found similar characteristics comparing digital empathy with that in the usual face-to-face setting (Friesem, 2016a,b; Terry and Cain, 2016). In particular, according to Friesem, digital empathy explores the ability […] to have a sense of identity and agency (self-empathy), the latter seems to be a specific feature of digital empathy and leads us to reflect: During a VCP session, the therapist, thanks to the web camera, can observe her/himself and her/his expressions, as well as the patient and her/his expressions, this fact makes the therapist more aware of his/her behaviors and expressions, which sharpens her/his awareness process in offering help to the other and probably increases her/his capability to be supportive, compassionate, and empathic.

It is surprising to note the fact that the percentage of concordance of the responses between patient and therapist to the two ESS subscales in remote sessions is significantly higher than the percentage of agreement found in face-to-face sessions. First of all, these data did not show any correlation with psychopathological aspects present in the patients. They describe the presence of a great therapeutic alliance between patients and psychotherapists in the online setting. It is generally known in the existing literature that the concordance index in the perception of empathy and support between patient and therapist is an element capable of predicting a good alliance between the two members of the couple as well as being predictive of a good outcome of the psychotherapeutic process. In our study, these data are really interesting and deserve further investigation. First of all, it allows us to affirm that VCP not only works but that it can be an adequate setting capable of promoting successful psychotherapy paths, in our study it even seems to work better than the face-to-face setting. Keeping aside for a moment the particular event created by the pandemic, it is certainly possible to say that adequate levels of empathy and support, which are functional to the success of the outcome, can also be achieved in a psychotherapeutic setting that involves the presence of a digital medium, such as a PC, a tablet, or smartphone. Therefore, even if the potential of online psychotherapy is still underestimated, we can say that online psychotherapy can be a good complement to face-to-face psychotherapy rather than a substitute for it (Longobardi et al., 2018).

Probably, in our study, the greater empathy and support perceived by patients can be explained by the fact that VCP allows a better and more channeled perception of those parameters other studies found to be fundamental to being empathic. Some of such parameters certainly include giving adequate attention to facial expressions and vocal intonation (Maldonato et al., 2018).

## Conclusion

This paper describes the results coming from the second step of an already implemented study which, in the first phase, evaluated the degree of satisfaction of a sample of Italian psychotherapists in the use of VCP during the COVID-19 emergency, in a condition that has never occurred in the history of psychotherapy research.

In the previous phase, the theoretical and methodological backgrounds were found to be elements capable of fostering the therapist’s perceived satisfaction using VCP.

For this reason, in this second phase, we hypothesized that the level of affective attunement and more widely the degree of empathy between the members of the psychotherapist-client dyad had specific characteristics and represented an efficacy factor for the success of the treatment, and also in the online setting.

Therefore, in order to discriminate the specific characteristics of digital empathy, we analyzed the degree of empathy between psychotherapist and client, as well as the degree of support perceived by the patient from his/her psychotherapist, through comparing the sessions in-person with those online, during the COVID-19 pandemic.

What emerged from comparing scores obtained by therapists and patients to the two subscales was amazing: Unlike the psychotherapists, the patients perceived their therapists as significantly more empathic and supportive in the remote setting.

These are rather important data, because the literature documents that client empathic perception measures represent a more accurate evaluation of the empathic relationship and, in general, can predict a good outcome.

Although these results need further investigation, they represent an important contribution in filling the scientific gap in the understanding of digital empathy. In fact, the characteristics and mechanisms underlying digital empathy are still too little studied and little known.

The innovation of this research is to highlight the real impact of digital empathy in the use of VCP, making it possible to obtain new contributions in an area that is still little known and investigated. We can conclude this study provides new insights for future research on the characteristics of empathy and the influence it has on the practice, the efficacy, and the good outcome of remote psychotherapy.

One of the limitations of the study, due to the still small size of the sample, concerns the impossibility of correlating the results relating to perceived empathy with individual aspects.

In particular, although we collected data relating to the digital setting (the quality and type of devices used, quality of internet connection, chosen location, etc.), the psychopathological characteristics, the personal predisposition of the subjects to be empathic, and the limited small size of the sample did not allow us to discriminate the significant differences between subjects regarding these variables.

In the literature, there are pieces of evidence about the fact that online therapy is more suitable for some types of patients than others (people with mobility problems, people with anxiety disorders, people who fear social stigma, people who have time constraints as managers or professionals, those who often move their residence for study or work reasons, those who are socially isolated for different reasons) (Longobardi et al., 2018; Cioffi et al., 2020), so it would be interesting to explore if and how these preferences could influence empathic perception.

Moreover, for future developments of this research, it might be a good idea to analyze the variability due to geographical and temporal differences in the experiences of the COVID-19 pandemic.

The current pandemic has made it necessary to change the setting of many therapeutic processes in progress. This study has collected the good satisfaction of patients in this change of setting in favor of the online one and certainly stimulates reflection on the opportunities that the online setting offers. The latter calls each psychotherapist to the challenge of adapting their clinical practice to changes in society, expanding the internal debate on the specificities of each model of remote work.

We intend to use these provisional results obtained from this first phase in the subsequent phases to explore further how empathy works in the online setting and what its specific features are, in order to improve the psychotherapists’ ability to exploit technologies and meet the psychological needs of clients in online settings.

In particular, to understand better which are the specific characteristics of the digital affective attunement process (Maldonato et al., 2017, 2018; Sperandeo et al., 2018), in the next step we intend to measure and compare the degree of tuning of psychophysiological parameters such as skin conductance, oxygen saturation, blood pressure, and heart rate. The detection of such psychophysiological parameters will take place through specific devices to obtain measurements both in in-person and remote settings.

## Data Availability Statement

The original contributions presented in the study are included in the article/Supplementary Material, further inquiries can be directed to the corresponding author.

## Ethics Statement

The studies involving human participants were reviewed and approved by Consiglio del Dipartimento 11/26.05.2020 Prot. n. 46540. The patients/participants provided their written informed consent to participate in this study.

## Author Contributions

RS, VC, EM, TL, YA, and LM contributed to conception and design of the study. VC organized the database. RS and VC performed the statistical analysis. DC, CG, MA, and RS wrote the first draft of the manuscript. BM, CS, and NM wrote sections of the manuscript. All authors contributed to manuscript revision, read, and approved the submitted version.

## Conflict of Interest

The authors declare that the research was conducted in the absence of any commercial or financial relationships that could be construed as a potential conflict of interest.

## Publisher’s Note

All claims expressed in this article are solely those of the authors and do not necessarily represent those of their affiliated organizations, or those of the publisher, the editors and the reviewers. Any product that may be evaluated in this article, or claim that may be made by its manufacturer, is not guaranteed or endorsed by the publisher.

## References

[B1] Åsberg M. Montgomery S. A. Perris C. Schalling D. Sedvall G. (1978). A comprehensive psychopathological rating scale. *Acta Psychiatr. Scand.* 57 5–27. 10.1111/j.1600-0447.1978.tb02357.x 277059

[B2] Backhaus A. Agha Z. Maglione M. L. Repp A. Ross B. Zuest D. (2012). Videoconferencing psychotherapy: a systematic review. *Psychol. Serv.* 9:111. 10.1037/a0027924 22662727

[B3] Barrett-Lennard G. T. (1986). “The relationship inventory now: Issues and advances in theory, method, and use,” in *The Psychotherapeutic Process: A Research Handbook*, eds lreenberg L. S. Pinsof W. M. (New York, NY: Guilford Press), 439–476.

[B4] Barrett-Lennard G. T. (2015). *The Relationship Inventory: A Complete Resource and Guide.* Hoboken, NJ: John Wiley & Sons.

[B5] Batson C. D. (2009). “These things called empathy: eight related but distinct phenomena,” in *The Social Neuroscience of Empathy*, eds Decety J. Ickes W. (Cambridge, MA: MIT Press), 3–16. 10.7551/mitpress/9780262012973.003.0002

[B6] Békés V. Aafjes-van Doorn K. (2020). Psychotherapists’ attitudes toward online therapy during the COVID-19 pandemic. *J. Psychother. Integr.* 30 238–247. 10.1037/int0000214

[B7] Berryhill M. B. Culmer N. Williams N. Halli-Tierney A. Betancourt A. Roberts H. (2019a). Videoconferencing psychotherapy and depression: a systematic review. *Telemed. J. E Health* 25 435–446. 10.1089/tmj.2018.0058 30048211

[B8] Berryhill M. B. Halli-Tierney A. Culmer N. Williams N. Betancourt A. King M. (2019b). Videoconferencing psychological therapy and anxiety: a systematic review. *Fam. Pract.* 36 53–63. 10.1093/fampra/cmy072 30188992

[B9] Cheshire W. P. Barrett K. M. Eidelman B. H. Mauricio E. A. Huang J. F. Freeman W. D. (2020). Patient perception of physician empathy in stroke telemedicine. *J. Telemed. Telecare.* 10.1177/1357633X19899237 [Epub ahead of print], 31986965

[B10] Cioffi V. Cantone D. Guerriera C. Architravo M. Mosca L. L. Sperandeo R. (2020). “Satisfaction degree in the using of VideoConferencing Psychotherapy in a sample of Italian psychotherapists during Covid-19 emergency,” in *Proceedings of the 11th IEEE International Conference on Cognitive Infocommunications (CogInfoCom)*, Mariehamn, 000125–000132. 10.1109/CogInfoCom50765.2020.9237823

[B11] Cipolletta S. Frassoni E. Faccio E. (2018). Construing a therapeutic relationship online: an analysis of videoconference sessions. *Clin. Psychol.* 22 220–229. 10.1111/cp.12117

[B12] Di Corrado D. Magnano P. Muzii B. Coco M. Guarnera M. De Lucia S. (2020). Effects of social distancing on psychological state and physical activity routines during the COVID-19 pandemic. *Sport Sci. Health* 16 619–624. 10.1007/s11332-020-00697-5 32994822 PMC7517049

[B13] Dolce P. Marocco D. Maldonato N. M. Sperandeo R. (2020). Toward a machine learning predictive-oriented approach to complement explanatory modeling. an application for evaluating psychopathological traits based on affective neurosciences and phenomenology. *Front. Psychol.* 2020:446. 10.3389/fpsyg.2020.00446 32265781 PMC7105860

[B14] Elliot R. Bohart A. C. Watson J. C. Grimberg L. S. (2002). “Empathy in Psychotherapy relationships that work,” in *Psychotherapy Relationships that Work: Therapist Contributions and Responsiveness to Patients*, ed. Norcross J. C. (Oxford: Oxford University Press).

[B15] Elliott R. Bohart A. C. Watson J. C. Greenberg L. S. (2011). Empathy. *Psychotherapy* 48:43. 21401273 10.1037/a0022187

[B16] Elliott R. Bohart A. C. Watson J. C. Murphy D. (2018). Therapist empathy and client outcome: an updated meta-analysis. *Psychotherapy (Chic.)* 55 399–410. 10.1037/pst0000175 30335453

[B17] Feller C. P. Cottone R. R. (2003). The importance of empathy in the therapeutic alliance. *J. Humanist. Couns. Educ. Dev.* 42 53–61. 10.1002/j.2164-490x.2003.tb00168.x

[B18] Friesem Y. (2016a). “Developing digital empathy: a holistic approach to media literacy research methods,” in *Handbook of Research on Media Literacy in the Digital Age*, Vol. 1 eds Yildiz M. N. Keengwe J. (Hershey, PA: IGI Global), 145–160. 10.4018/978-1-4666-9667-9.ch007

[B19] Friesem Y. (2016b). “Empathy for the digital age: using video production to enhance social, emotional, and cognitive skills,” in *Emotions, Technology, and Behaviors*, eds Tettegah S. Espelage D. L. (Cambridge, MA: Academic Press), 21–45.

[B20] Grummon D. L. (1972). Different approaches to the measurement of therapist empathy and their relationship to therapy outcomes. *J. Consult. Clin. Psychol.* 39 106–115. 10.1037/h0033190 5045268

[B21] Longobardi T. Sperandeo R. Albano F. Tedesco Y. Moretto E. Di Sarno A. D. (2018). “Co-regulation of the voice between patient and therapist in psychotherapy: machine learning for enhancing the synchronization of the experience of anger emotion: An experimental study proposal,” in *Proceedings of the 2018 9th IEEE International Conference on Cognitive Infocommunications (CogInfoCom)*, Budapest, 000113–000116. 10.1109/CogInfoCom.2018.8639875

[B22] Maldonato N. M. Bottone M. Sperandeo R. Scandurra C. Bochicchio V. Cappuccio M. L. (2020). “The biodynamic stress hypothesis Towards an evolutionary psychology paradigm,” in *Proceedings of the 2019 10th IEEE International Conference on Cognitive Infocommunications (CogInfoCom)*, Naples, 369–374. 10.1109/CogInfoCom47531.2019.9089930

[B23] Maldonato N. M. Sperandeo R. Dell’Orco S. Cozzolino P. Fusco M. L. Iorio V. S. (2017). The relationship between personality and neurocognition among the American elderly: an epidemiologic study. *Clin. Pract. Epidemiol. Mental Health* 13 233–245. 10.2174/1745017901713010233 29299046 PMC5725479

[B24] Maldonato N. M. Sperandeo R. Moretto E. Dell’Orco S. (2018). A non-linear predictive model of borderline personality disorder based on multilayer perceptron. *Front. Psychol.* 9:447. 10.3389/fpsyg.2018.00447 29670562 PMC5893824

[B25] Meador B. D. Rogers C. R. (1984). Person-centered therapy. *Curr. Psychother.* 2 131–184.

[B26] Messina I. Sambin M. Palmieri A. (2013). Measuring therapeutic empathy in a clinical context: validating the Italian version of the empathic understanding of relationship inventory. *TPM Test. Psychometr. Methodol. Appl. Psychol.* 20 1–11.

[B27] Nascivera N. Alfano Y. M. Annunziato T. Messina M. Iorio V. S. Cioffi V. (2018). “Virtual empathy: the added value of virtual reality in psychotherapy,” in *Proceedings of the 2018 9th IEEE International Conference on Cognitive Infocommunications (CogInfoCom)*, Budapest, 000321–000326. 10.1109/CogInfoCom.2018.8639906

[B28] Norwood C. Moghaddam N. G. Malins S. Sabin-Farrell R. (2018). Working alliance and outcome effectiveness in videoconferencing psychotherapy: a systematic review and noninferiority meta-analysis. *Clin. Psychol. Psychother.* 25 797–808. 10.1002/cpp.2315 30014606

[B29] Reese R. J. Mecham M. R. Vasilj I. Lengerich A. J. Brown H. M. Simpson N. B. (2016). The effects of telepsychology format on empathic accuracy and the therapeutic alliance: an analogue counselling session. *Couns. Psychother. Res.* 16 256–265. 10.1002/capr.12092

[B30] Reilly S. E. Zane K. L. McCuddy W. T. Soulliard Z. A. Scarisbrick D. M. Miller L. E. (2020). Mental health practitioners’ immediate practical response during the COVID-19 pandemic: observational questionnaire study. *JMIR Ment. Health* 7:e21237. 10.2196/21237 32931440 PMC7546864

[B31] Simpson S. Richardson L. Pietrabissa G. Castelnuovo G. Reid C. (2020). Videotherapy and therapeutic alliance in the age of COVID−19. *Clin. Psychol. Psychother.* 28 409–421. 10.1002/cpp.2521 33037682 PMC7675483

[B32] Simpson S. G. Reid C. L. (2014). Therapeutic alliance in videoconferencing psychotherapy: a review. *Aust. J. Rural Health* 22 280–299. 10.1111/ajr.12149 25495622

[B33] Sperandeo R. Longobardi T. Mosca L. L. Cioffi V. Moretto E. Alfano Y. M. (2020). “Psychotherapy in interaction with Technology: Proposal of a new investigation area for CogInfoCom,” in *Proceedings of the 2020 11th IEEE International Conference on Cognitive Infocommunications (CogInfoCom)*, Mariehamn, 000321–000324. 10.1109/CogInfoCom50765.2020.9237811

[B34] Sperandeo R. Picciocchi E. Valenzano A. Cibelli G. Ruberto V. Moretto E. (2018). Exploring the relationships between executive functions and personality dimensions in the light of embodied cognition theory: a study on a sample of 130 subjects. *Acta Med. Mediterr.* 34 1271–1279. 10.19193/0393-6384_2018_5_196

[B35] Terry C. Cain J. (2016). The emerging issue of digital empathy. *Am. J. Pharm. Educ.* 80:58. 10.5688/ajpe80458 27293225 PMC4891856

[B36] Weinberg H. (2021). Obstacles, challenges, and benefits of online group psychotherapy. *Am. J. Psychother.* 74 83–88. 10.1176/appi.psychotherapy.20200034 33525914

