# Supplementary material for: Exploring the Question: “Does Empathy Work in the Same Way in Online and In-Person Therapeutic Settings?”
Source: Front Psychol. 2021 Sep 21;12:671790. doi: 10.3389/fpsyg.2021.671790 (PMC8490728; doi:10.3389/fpsyg.2021.671790)
Supplement: Supplementary file 1 [file Table_1.docx]

**Table 1 - Eight empathy phenomena**

| **Phenomena** | **Definition** | **Scholars** |
| --- | --- | --- |
| **Cognitive empathy** | “Empathic accuracy”, knowing another person’s internal state, including his or her thoughts and feelings | Ickes (1997) and Wispe´ (1986) |
| **Facial empathy** | Adopting the posture or matching the neural responses of an observed other | Gordon (1995) and Meltzoff and Moore (1997) |
| **Affective empathy** | “Emotional contagion”, coming to feel as another person feels | Hatfield, Cacioppo, and Rapson (1994) and ZahnWaxler, Robinson, and Emde (1992) |
| **Aesthetic empathy** | Intuiting or projecting oneself into another’s situation | Wispe´ (1968) |
| **Psychological**  **Empathy** | “Perspective taking” or “empathic attention set”, imagining how another is thinking and feeling | Barrett-Lennard (1981), Ruby and Decety (2004), and Stotland (1969) |
| **Projective empathy** | “Role taking”, imagining how one would think and feel in the other’s place | Darwall (1998) and Mead (1962) |
| **Empathic distress** | Feeling distressed at witnessing another person’s suffering | Hoffman (1981) |
| **Empathic concern** | “Sympathy”, feeling for another person who is suffering | Batson (1991) and Preston and de Waal (2002) |

Source: Adapted from Batson, C. D. (2009). These things called empathy: Eight related but distinct phenomena. In J. Decety, & W. Ickes (Eds.), The social neuroscience of empathy (pp. 3-16). Cambridge, MA: MIT Press.
