# Supplementary material for: Exploring the Question: “Does Empathy Work in the Same Way in Online and In-Person Therapeutic Settings?”
Source: Front Psychol. 2021 Sep 21;12:671790. doi: 10.3389/fpsyg.2021.671790 (PMC8490728; doi:10.3389/fpsyg.2021.671790)
Supplement: Supplementary file 2 [file Table_2.docx]

**Table 2 - Socio-demographic and clinic characteristics of the sample (N = 24)**

|  | N |  | N |
| --- | --- | --- | --- |
| **Patients** | 24 | **Psychotherapists** | 5 |
| **Men** | 5 | **Men** | 2 |
| **Women** | 19 | **Women** | 3 |
| **Age (A ± SD)** | 36.35 ± 9.09 | **Age (A ± SD)** | 47.17 ± 6.13 |
| **Psychic disorders of mild severity** | 7 | **Psychoanalytic approach** | 2 |
| **Psychic disorders of moderate severity** | 6 | **Gestaltic approach** | 2 |
| **Without current mental disorders** | 11 | **Transactional analysis approach** | 1 |
| **In-person sessions** | 33 | **Online sessions** | 39 |
