# Supplementary material for: Exploring the Question: “Does Empathy Work in the Same Way in Online and In-Person Therapeutic Settings?”
Source: Front Psychol. 2021 Sep 21;12:671790. doi: 10.3389/fpsyg.2021.671790 (PMC8490728; doi:10.3389/fpsyg.2021.671790)
Supplement: Supplementary file 3 [file Table_3.docx]

**Table 3 - Empathy and support assessed in both patients and psychotherapists**

|  | **Psychotherapists** | | | | **Patients** | | | |
| --- | --- | --- | --- | --- | --- | --- | --- | --- |
|  | **Online sessions** | **In-person sessions** | **T** | **P** | **Online sessions** | **In-person sessions** | **t** | **P** |
| **ESPS**  **A (SD)** | 38.84 (3.88) | 38.76 (4.37) | 0.09 | N.S. | 40.44 (3.02) | 37.42 (5.26) | 2.82 | < 0.01 |
| **ESNS**  **A (SD)** | 1.84 (2.39) | 2.27 (3.08) | 0.66 | N.S. | 1.56 (3.20) | 3.24 (3.64) | 2.00 | < 0.04 |

Comparison between the group of subjects treated online and those treated in person. Student's T-test among the means of the scores obtained on the empathy and support subscales.
