# Supplementary material for: Exploring the Question: “Does Empathy Work in the Same Way in Online and In-Person Therapeutic Settings?”
Source: Front Psychol. 2021 Sep 21;12:671790. doi: 10.3389/fpsyg.2021.671790 (PMC8490728; doi:10.3389/fpsyg.2021.671790)
Supplement: Supplementary file 4 [file Table_4.docx]

**Table 4 - Agreement between patients and therapists in the perception of empathy and support**

|  | **Number of concordant observations** | **Number of non concordant observations** | **% of concordant observations** | **X^2^** | **P** |
| --- | --- | --- | --- | --- | --- |
| **Face-to-face ESPS** | 282 | 166 | 62.9 | 6.59 | 0.01 |
| **Digital ESPS** | 376 | 156 | 70.7 |  |  |
| **Face-to-face ESNS** | 321 | 127 | 71.7 | 17.77 | 0.00 |
| **Digital ESNS** | 441 | 91 | 82.9 |  |  |

Comparison between the concordance between patients and therapists in face-to-face and remote sessions. Test X2 applied to the number of observations made in-person and online.
