# Supplementary material for: Exploring the Question: “Does Empathy Work in the Same Way in Online and In-Person Therapeutic Settings?”
Source: Front Psychol. 2021 Sep 21;12:671790. doi: 10.3389/fpsyg.2021.671790 (PMC8490728; doi:10.3389/fpsyg.2021.671790)
Supplement: Supplementary file 5 [file Table_5.docx]

***Support and Empathy Scale***

*Form for the psychotherapist – ESS-P*

Legga le affermazioni che seguono e, in relazione alta seduta odierna, indichi quanto concorda con ciascuna di esse, mettendo una crocetta nella casella corrispondente. (versione per lo psicoterapeuta)

0 = per niente/mai 1 = solo un po'/quasi mai 2 = abbastanza/a volte 3 = molto/spesso

Items positivi

1. Mi sono sentito affidabile per lui/lei
2. Ho percepito il rispetto, la dignità e il suo valore come persona
3. Sono stato accogliente e caldo nei suoi confronti
4. Ho espresso autenticamente le mie impressioni e i miei sentimenti
5. Mi sono sentito coinvolto, interessato e rispettoso verso i suoi sentimenti e i suoi bisogni
6. Ho sentito una piena comprensione dei suoi sentimenti
7. Ho saputo infondere un senso di fiducia verso il futuro
8. Abbiamo lavorato Insieme per arrivare a individuare gli elementi centrali del suo problema
9. Ho sentito di accettare profondamente il suo modo di essere
10. Ho detto esattamente quello che pensavo
11. Ho cercato di comprendere il suo punto di vista
12. Ho percepito fiducia nelle sue potenzialità
13. Ho facilitato la sua libera espressione con i miei atteggiamenti (postura, voce...)
14. Mi è sembrato di partecipare attivamente alla ricerca di soluzioni per i suoi problemi

Items negativi

1. Non ho saputo infondere un senso di fiducia verso il futuro
2. Non sempre ho compreso pienamente il suo punto di vista
3. Mi è sembrato di trascurare II suo bisogno di essere accolto
4. A volte posso essere stato/a critico/a, giudicante, con un atteggiamento di superiorità
5. Non mi sono sentito completamente sincero nell'espressione delle mie emozioni e sentimenti
6. Ho avuto dei dubbi sulle sue possibilità di riuscita
7. In alcuni momenti mi sono sentito poco coinvolto e interessato verso i suoi bisogni e sentimenti
8. Mi sono sentito poco partecipe alla ricerca di soluzioni per i suoi problemi
9. Ho detto cose che non pensavo veramente
10. Posso averlo fatto sentire poco considerati) e rispettato
11. Il mio atteggiamento (postura, voce...) posso aver ostacolato la comunicazione
12. Nella seduta di oggi abbiamo affrontata aspetti marginali del problema
13. Non mi sono sentito costantemente affidabile per lui/lei
14. l'impressione di non aver compreso come si sentiva profondamente

*Form for the client – ESS-C*

Legga le affermazioni che seguono e, in relazione alla seduta odierna, indichi quanto concorda con ciascuna di esse, mettendo una crocetta nella casella corrispondente. (Versione per l’utente)

0 = per niente/mai 1= solo un po'/quasi mai 2 =abbastanza/a volte 3 = molto/spesso

Items positivi

1. Ho sentito che potevo fidarmi di lui/lei
2. Mi ha fatto sentirà rispettato e degno di considerazione
3. E' stato accogliente e caldo nel miei confronti
4. Ha espresso autenticamente te sue impressioni e i suoi sentimene
5. L’ ho percepito coinvolto ed interessato verso i miei sentimenti ed i miei bisogni
6. Ha colto in pieno quello che sentivo
7. Dopo la seduta ho provato un senso di speranza verso il mio futuro
8. Sento che abbiamo lavorato insieme per arrivare a Individuare gli elementi centrali del mio problema
9. Ho potuto esprimermi senza timore di essere criticato o giudicato
10. Ho sentito che diceva esattamente quello che pensava
11. Ha cercato di comprendere il mio punto di vista
12. Ho percepito la sua fiducia nelle mie potenzialità
13. Il suo atteggiamento (postura, voce) ha facilitato la mia libera espressione
14. Mi è sembrato che partecipasse attivamente alla ricerca di soluzioni per i miei problemi

Items negativi

1. Dopo la seduta mi sono sentito sfiduciato verso il mio futuro
2. Non sempre ha compreso il mio punto di vista
3. Mi è sembrato che trascurasse il mio bisogno di accoglienza
4. Ha avuto un atteggiamento di superiorità, mi sono sentito criticato e giudicato
5. A volte quello che manifestava esternamente mi sembrava diverso da quello che sperimentava
6. Mi è sembrato che dubitasse delle mie possibilità di riuscita
7. L'ho sentito distaccato e disinteressato verso i miei bisogni ed i miei sentimenti
8. Mi è sembrato distante dalla ricerca di soluzioni per i miei problemi
9. Quello che diceva mi sembrava diverso da quello che pensava
10. Mi sono sentito poco considerato e rispettato
11. Il suo atteggiamento mi ha reso difficile esprimermi liberamente
12. Nella seduta di oggi abbiamo affrontato aspetti del mio problema che considero marginali
13. in certi momenti mi è sembrato di non potermi fidare di lui/lei
14. Ha frainteso come mi sentivo profondamente

***Support and Empathy Scale (English Translation)***

*Form for the psychotherapist – ESS-P*

Read the following statements and, in relation to today's session, indicate how much you agree with each of them, by putting a cross in the corresponding box. (version for the psychotherapist)

0 = not at all / never 1 = only a little / hardly ever 2 = enough / sometimes 3 = very / often

Positive Items

1. I felt trustworthy [sic] for him/her

2. I perceived respect, dignity, and his value as a person

3. I have been welcoming and warm towards him

4. I have authentically expressed my impressions and feelings

5. I felt involved, interested, and respectful of his feelings and needs

6. I felt a full understanding of his feelings

7. I was able to instill a sense of confidence in the future

8. We worked together to identify the central elements of your problem

9. I felt deeply accepting [sic] his way of being

10. I said exactly what I thought

11. I tried to understand your point of view

12. I felt confident in its potential

13. I facilitated his free expression with my attitudes (posture, voice ...)

14. It seemed to me that I was actively involved in finding solutions to his problems

Negative Items

15. I have not been able to instill a sense of confidence in the future

16. I have not always fully understood your point of view

17. I seemed to overlook his need to be accepted

18. At times I may have been critical, judgmental, with an attitude of superiority

19. I have not felt completely sincere in the expression of my emotions and feelings

20. I had doubts about his chances of success

21. In some moments I felt a [sic] little involved and interested in his needs and feelings

22. I felt little involved in the search for solutions to his problems

23. I've said things I didn't really mean

24. I may have made him feel little considered and respected

25. My attitude (posture, voice ...) may have hindered communication

26. In today's session we have dealt with marginal aspects of the problem

27. I have not consistently felt reliable for him/her

28. The impression of not having understood how deeply he felt

*Form for the client ESS-C*

Read the following statements and, in relation to today's session, indicate how much you agree with each of them, by putting a cross in the corresponding box. (Client version)

0 = not at all / never 1 = only a little / hardly ever 2 = enough / sometimes 3 = very / often

Positive Items

1. I felt that I could trust him/her

2. It made me feel respected and worthy of consideration

3. He was welcoming and warm towards me

4. He has authentically expressed his impressions and feelings

5. I perceived him as involved and interested in my feelings and needs

6. He fully grasped what I was feeling

7. After the session, I felt a sense of hope for my future

8. I feel we have worked together to get to [sic] identify the core elements of my problem

9. I was able to express myself without fear of being criticized or judged

10. I heard he said exactly what he thought

11. He tried to understand my point of view

12. I felt his confidence in my potential

13. His attitude (posture, voice) facilitated my free expression

14. It seemed to me that he actively participated in finding solutions to my problems

Negative Items

1. After the session, I felt disheartened about my future

2. He didn't always understand my point of view

3. It seemed to me that he neglected my need for hospitality

4. He had an attitude of superiority: I felt criticized and judged

5. Sometimes, what he externally manifested seemed different to me from what he experienced

6. It seemed to me that he doubted my chances of success

7. I felt him detached and disinterested in my needs and my feelings

8. It seemed a long way from finding solutions to my problems

9. What he said seemed different to me than what he thought

10. I felt little considered and respected

11. Your attitude made it difficult for me to express myself freely

12. In today's session, we have addressed aspects of my problem which I consider marginal

13. At certain times, it seemed to me that I could not trust him/her

14. He misunderstood how deeply I felt
