# Supplementary figures and images for: Exploring the Question: “Does Empathy Work in the Same Way in Online and In-Person Therapeutic Settings?”
Source: Front Psychol. 2021 Sep 21;12:671790. doi: 10.3389/fpsyg.2021.671790 (PMC8490728; doi:10.3389/fpsyg.2021.671790)

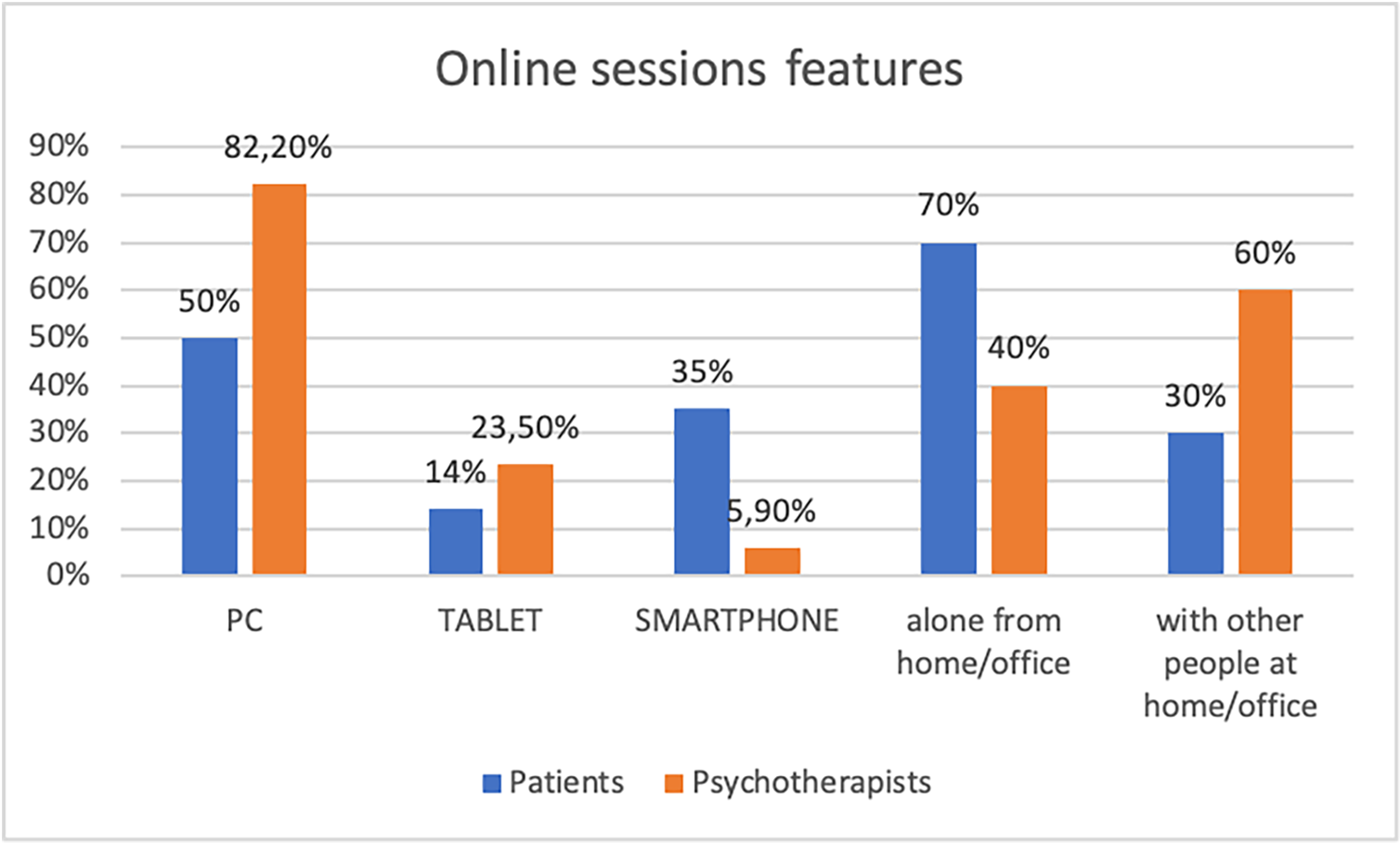

Supplement: Supplementary file 6 [file Image_1.png]
